# SDF-1 expression and tumor-infiltrating lymphocytes identify clinical subtypes of triple-negative breast cancer with different responses to neoadjuvant chemotherapy and survival

**DOI:** 10.3389/fimmu.2022.940635

**Published:** 2022-10-20

**Authors:** Ruo-Xi Wang, Peng Ji, Yue Gong, Zhi-Ming Shao, Sheng Chen

**Affiliations:** ^1^ Department of Breast Surgery, Cancer Institute, Fudan University Shanghai Cancer Center, Shanghai, China; ^2^ Department of Oncology, Shanghai Medical College, Fudan University, Shanghai, China; ^3^ Institutes of Biomedical Science, Fudan University, Shanghai, China

**Keywords:** breast cancer, neoadjuvant chemotherapy, SDF-1, TILs, pathological complete response

## Abstract

**Background:**

In this study, we investigated the prediction and prognostic value of SDF-1 for triple-negative breast cancer (TNBC) patients who underwent neoadjuvant chemotherapy (NAC) following standard radical surgery.

**Methods:**

A total of 303 TNBC patients were included in this study. The NAC regimen was weekly paclitaxel plus carboplatin (PC) for all patients. SDF-1 and CXCR4 expression were measured at baseline and surgery *via* enzyme-linked immunosorbent assay (ELISA) and immunohistochemistry (IHC), respectively. Correlations between variables and treatment response were studied, and Cox proportional hazards regression analysis was implemented for prognostic evaluation.

**Results:**

Of the 303 patients, 103 (34.0%) experienced pathological complete response (pCR) after completion of NAC. Serum SDF-1 expression before NAC was significantly correlated with the abundance of TILs. A higher pCR rate was more likely to be observed in patients with lower serum SDF-1 levels before NAC (P=0.001, OR=0.997, 95% CI: 0.996-0.999) and higher levels of TILs (P=0.005). In the multivariate survival model for nonpCR patients, serum SDF-1 expression at surgery served as an independent prognostic value for survival (high level, HR=1.980, 95% CI: 1.170-3.350, low level was used as a reference; P=0.011). Additionally, the predictive and prognostic value of serum SDF-1 expression was significant in patients with high abundance of TILs but not in patients with low abundance of TILs.

**Conclusions:**

This study contributes to the clarification of the value of serum SDF-1 to predict pCR and survival for TNBC patients who underwent NAC. This new serum marker, together with TILs, might help identify clinical subtypes of TNBC with different treatment responses and survival and play an important role in tailoring and modifying the NAC strategy for advanced TNBCs in the future.

## Background

Triple-negative breast cancer (TNBC) is a type of breast cancer that exhibits low expression of estrogen receptor (ER), progesterone receptor (PgR), and human epidermal growth factor receptor-2 (HER2) (1). TNBC accounts for 15-20% of all breast cancers and has an aggressive tumor biology. Neoadjuvant chemotherapy (NAC), also known as preoperative chemotherapy, followed by definitive surgery is a standard of care for locally advanced TNBC and early-stage TNBC with relatively large tumor sizes. The outcome of NAC is usually assessed based on the pathological response of surgical specimens and has a significant impact on patient survival. Patients who achieve a pathological complete response (pCR) have a relatively lower risk of disease recurrence or death than patients with residual disease after NAC (2, 3). Although the

definition of pCR varies across different studies, it has been accepted that the ideal definition should be absence of invasive cancer within both breast and nodes. In earlier studies, analyses were performed based on biological variables (such as ER, PR) through classical cutoffs to predict pCR, however, new biomarkers with more sensitivity and accuracy for early prediction of pCR are still needed.

TNBC is a heterogeneous disease comprising multiple subtypes with different biological behaviors and clinical outcomes (4). However, due to clinical accessibility and convenience, the genomic features of TNBC are still not mature enough to enable the prediction of treatment response. Recent studies have reported numerous biomarkers (e.g., tumor size, node status, Ki-67, HER2) and imaging-based metrics (e.g., magnetic resonance imaging [MRI] and positron emission tomography) for the prediction of pCR (5, 6); however, most efforts with traditional biomarkers measured prior to chemotherapy lack accuracy, and most efforts focusing on monitoring changes in morphological characteristics are indicative only of a late-stage response (7–10).

Stromal cell-derived factor-1 (SDF-1), also known as CXC motif chemokine ligand-12 (CXCL12), which binds to the CXC receptors 4 and 7, is ubiquitously expressed in almost all organs and involved in several aspects of tumor progression, including angiogenesis, metastasis, and survival (11). Some studies have shown that high expression of SDF-1 in cancer cells attracts CXCR4-positive cells, such as cancer-associated fibroblasts (CAFs) or tumor infiltrating lymphocytes (TILs), to the tumor sites and converts the tumor microenvironment (TME) to immune tolerance (12–14). Since TILs are a reliable marker of chemotherapy efficacy and are associated with clinical outcomes in breast cancer (15–17), it is plausible that SDF-1 might also play an essential role in the response to NAC according to environment-mediated drug resistance.

In this study, we analyzed the correlation of SDF-1 and TILs at different time points during NAC and aimed to demonstrate the predictive and prognostic performance of SDF-1 in chemo-naive and chemo-resistant TNBC.

## Patients and methods

### Study population

We retrospectively collected data from 303 patients with TNBC for this study according to inclusive and exclusive criteria reported in previous studies (18). TNBC was defined as ER-, PgR-, and HER2−. The cutoff values for ER positivity and PgR positivity were 1% of positive tumor cells with nuclear staining. HER2 was evaluated as 0, 1+, 2+, or 3+ using circumferential membrane-bound staining. Positivity for HER2 (HER2+) was considered as 3 + using immunohistochemistry (IHC) or as positive on fluorescence *in situ* hybridization (FISH), whereas cases with 0 to 1 + or 2 + using IHC but without FISH detection were regarded as negative for HER2 (HER2−). All patients were treated with six cycles of weekly PC (paclitaxel [80 mg/m^2^] and carboplatin [AUC 2 mg*min/ml] on Days 1, 8, and 15 of a 28-day cycle) followed by surgical resection of the primary breast and axillary lymph node at Shanghai Cancer Hospital between January 2009 and July 2015. Subsequently, patients with pCR received two additional cycles of the same regimen, whereas those who failed to reach pCR received three cycles of anthracycline-containing chemotherapy. Radiation therapy was performed at the discretion of the treating radiologist and was based on disease status before NAC. Patients treated with any other pre-operative treatment including radiotherapy, target therapy, endocrine therapy or chemotherapy were excluded from the study.

### Response and survival evaluation

pCR was defined as no residual invasive cancer in either the breast or lymph nodes. Patients with ductal carcinoma *in situ* (DCIS) only were also considered pCR responders. Patients were followed up every three months in the first two years after the operation and every six months after the first two years after the operation. Disease-free survival was calculated from the date of surgery to the date of disease relapse (local or distant relapse or death from any cause). Patients without events or death were censored at the last follow-up.

### ELISA and immunohistochemistry

**Figure 1:**
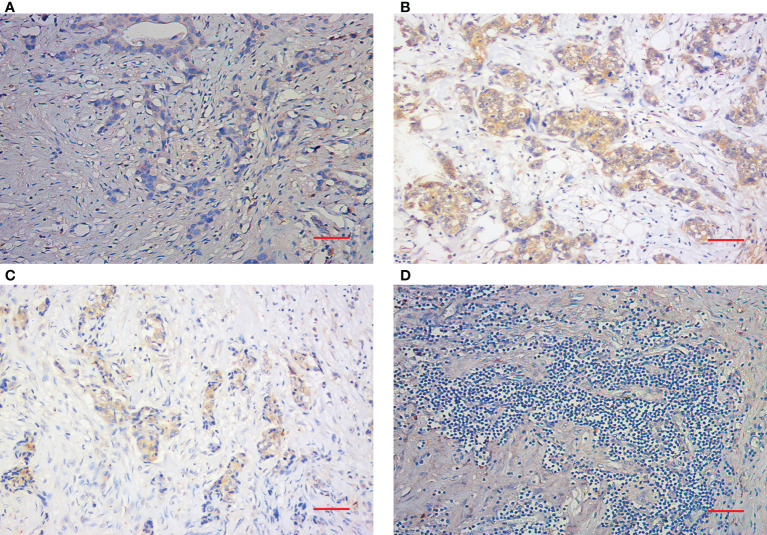
Immunohistochemical staining of TILs and CXCR4. **(A)** Representative IHC images of negative CXCR4 staining (200X). **(B)** Representative IHC images of positive CXCR4 staining. **(C)** Representative IHC images of low TIL staining. **(D)** Representative IHC images of high TIL staining (200X). Scale bar (Red): 50 μm.

Peripheral blood samples were collected prior to the start of NAC (at baseline) and after the completion of NAC (at surgery). Blood samples were centrifuged at 1800 rpm for 15 min at 4°C, and serum was transferred to tubes and stored at −80°C until the time of analysis. The serum SDF-1 levels were blindly evaluated concurrently by using a quantitative sandwich ELISA kit (RAB0123-1KT, Sigma–Aldrich) according to a standard protocol. For each serum sample, measurement was repeated at three time points, and the final result was marked as the average level. The inter- and intra-assay coefficients of variation for SDF-1 were 3.5–5.2% and 3.3–6.2%, respectively. The detection range was 93.75-6000 pg/mL. IHC was performed on formalin-fixed, paraffin-embedded tissue sections collected from core-needle biopsy and residual tumor specimens using a two-step protocol (GTVisionIII) to evaluate CXCR4 expression. The antibody was purchased from Cell Signaling Technology (AB124824, Abcam). As a negative control, the primary antibody was omitted and replaced by 1% BSA-PBS. The immunostained slides were evaluated independently by two pathologists. The H score was used to define the positivity of variables (19). An H score of < 100 was defined as negative, whereas an H score > 100 was considered positive. The assessment of unstained TILs was based on the recommendation of an International TIL Working Group (20). TILs were evaluated within the stromal compartment close to the invasive tumor and reported as the percentage of stromal TILs. Representative pathological images (200X) are shown in **Figure 1**.

### Statistical analysis

The two-tailed Student’s T test was used to compare differences in SDF-1 expression between the two groups. The chi-squared test was used to evaluate the relationships between patient characteristics and pathological response. Variables that significantly predicted pCR in the chi-square test were entered into the multivariate analyses using a logistic regression model. Univariate and multivariate survival analyses were performed using the Cox regression model. Survival curves were estimated using the Kaplan–Meier method, and the log-rank test was used to test for differences between groups. All statistical tests were two-sided, and P values less than 0.05 were considered significant. All analyses were performed with SPSS (version 19.0, SPSS Company, Chicago, IL, USA).

## Results

### Patient characteristics

**Table 1** shows the main characteristics of all patients. The median age of the 303 patients was 50 years (range, 27-74 years). A total of 159 patients were premenopausal at diagnosis, whereas 144 patients were postmenopausal. All patients were diagnosed with T stage between T2-T4, whereas 82.8% of all patients had positive nodes before NAC. TILs in the stromal area of the tumor bed were counted according to the recommendation by an International TIL Working Group (20), whereas TILs outside of the tumor border were excluded. Patients were classified into the high TIL group with the recommended cutoff of 50%. A total of 133 patients (43.9%) had high levels of TILs, and 170 patients (56.1%) had low levels of TILs. Serum SDF-1 expression was detected according to ELISA prior to NAC. The median level was 329.0 pg/ml (range: 100.0 pg/ml-1158.3 pg/ml). A total of 186 patients were identified as CXCR4 positive through IHC, whereas the remaining 117 patients were CXCR4 negative. **Supplemental Figure 1** shows the distribution of SDF-1 expression among different characteristics. SDF-1 expression was similar in patients with different ages, menopausal statuses, tumor stages, and Ki67 levels; however, high expression of SDF-1 was observed in patients with low levels of TILs (mean level 376.3 ± 188.1 vs. 315.3 ± 182.7, P=0.0048) and in patients with high expression of CXCR4 (mean level 295.4 ± 161.3 vs. 383.6 ± 195.7, P<0.001).

**Table 1:** Patient characteristics and observed pathological complete response (pCR).

Characteristics	Number of patients (%)	Number of pCR (%)	Chi-Square P value	Multivariate P value	Exp. OR (95%CI)
Age			0.564	–	
<40	60 (19.8)	23 (38.3)			
40-59	194 (64.0)	66 (34.0)			
60+	49 (16.2)	14 (28.6)			
Menopausal status			0.817	–	
Pre	159 (52.5)	55 (34.6)			
Post	144 (47.5)	48 (33.3)			
Tumor stage			0.026	0.034	
T2	150 (49.5)	62 (41.3)			Ref.
T3	100 (33.0)	28 (28.0)			0.510 (0.275-0.947)
T4	53 (17.5)	13 (24.5)			0.449 (0.210-0.962)
Node status			0.917	–	
–	52 (17.2)	18 (34.6)			
+	251 (82.8)	85 (33.9)			
TILs			<0.001	<0.001	
<50%	170 (56.1)	41 (24.1)			Ref.
≥50%	133 (43.9)	62 (46.6)			4.607 (1.530-4.442)
Ki-67 expression			<0.001	0.001	
<20%	107 (35.3)	22 (20.6)			Ref.
≥20%	196 (64.7)	81 (41.3)			2.618 (1.456-4.707)
Serum SDF-1 (pg/ml)			<0.001	0.005	
<200.0	68 (22.4)	36 (52.9)			Ref.
200-299.9	55 (18.2)	23 (41.8)			0.515 (0.233-1.136)
300-399.9	69 (22.8)	19 (27.5)			0.304 (0.142-0.650)
400-499.9	51 (16.8)	13 (25.5)			0.318 (0.136-0.743)
≥500	60 (19.8)	12 (20)			0.264 (0.114-0.609)
CXCR4 expression			0.421	–	
Negative	117 (38.6)	43 (36.8)			
Positive	186 (61.4)	60 (32.3)			

pCR, pathological complete response; OR, odds ratio; CI, confidence interval; TILs, tumor-infiltrating lymphocytes; SDF-1, stromal cell-derived factor-1; Ref., reference.

### Variables that predict pCR

**Figure 2:**
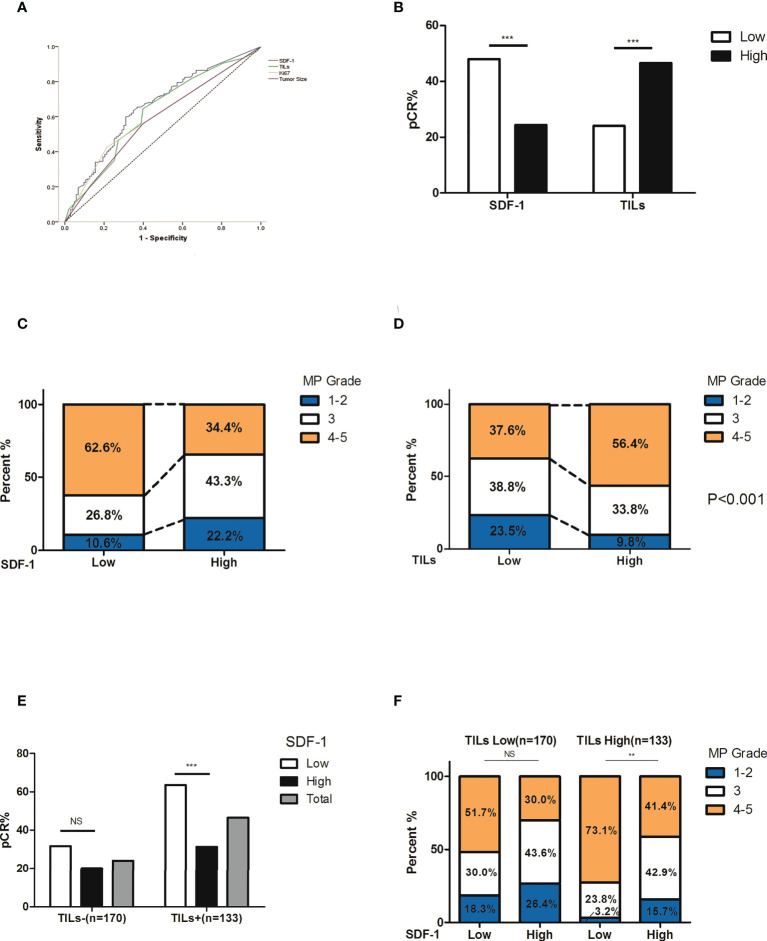
Correlation between treatment response and its predictors. **(A)** ROC curve of pCR predictors. The AUCs were 0.657, 0.624, 0.606, and 0.585 for SDF-1, TILs, Ki67, and T stage, respectively. **(B)** The rate of pCR and SDF-1 expression. The pCR rate was 48.0% in patients with low SDF-1 expression and 24.4% in patients with high SDF-1 expression (P<0.001). **(C)** The correlation between the SDF-1 levels and tumor regression (according to MP grades). Notably, there was a higher proportion of favorable responses in SDF-1-low patients. **(D)** The correlation between TILs and tumor regression (according to MP grades). Notably, there was a higher proportion of favorable responses in SDF-1 high expression patients. **(E)** The correlation between SDF-1 and pCR according to different TIL levels. The pCR rate in patients with high TILs was 63.5% for low SDF-1 and 31.4% for high SDF-1 (P<0.001). The pCR rate in patients with low TILs was 31.7% for low SDF-1 and 20.0% for high SDF-1 (P=0.089). **(F)** The correlation between SDF-1 and tumor regression (according to MP grades) according to different TIL levels. Notably, patients with high TILs and low SDF-1 levels experienced a favorable response to NAC. (NS, not significant; **P<0.01, ***P<0.001).

Among the 303 patients, the overall pCR rate was 34.0% (103/303). The correlations between multiple patient characteristics and pCR were analyzed (**Table 1**). In univariate analysis, the primary T stage (P=0.026), TILs (P<0.001), Ki-67 expression (P<0.001), and serum SDF-1 (P<0.001) were identified as pCR predictors, and we found no significant differences in pCR according to patient age, menopausal status, node status, and CXCR4 expression. In the multivariate logistic regression model, SDF-1 was independently correlated with pCR (P=0.005). TILs were also independently correlated with pCR (P<0.001). The OR of TILs ≥50% was 4.607 (95% CI: 1.530-4.442, P<0.001, TILs <50% as reference). Tumor T stage and Ki-67 expression were also independent predictors of pCR (P=0.034, OR=0.510 for T3, and OR=0.449 for T4, T2 as reference; and P=0.001, HR=2.618 for high Ki67, low Ki-67 as reference, respectively). The ROC curves of the pCR predictors are shown in **Figure 2A**. The AUCs were 0.657, 0.624, 0.606, and 0.585 for SDF-1, TILs, Ki67, and T stage, respectively.

### Serum SDF-1, TILs, and treatment response

The pCR rates were 52.9%, 41.8%, 27.5%, 25.5%, and 20% according to different SDF-1 levels (<200.0 pg/ml, 200-299.9, 300-399.9 pg/ml, 400-499.9 pg/ml, and ≥500.0 pg/ml, respectively). A higher pCR possibility was more likely observed in patients with lower levels of SDF-1, especially in patients with SDF-1 expression of <300.0 pg/ml. According to the ROC curve of SDF-1 (AUC=0.657, 95% CI: 0.592-0.722), the cutoff value of SDF-1 to predict pCR was 328.25 pg/ml, with the largest sum of sensitivity and specificity. For the sake of convenience, we set the cutoff as 300.0 pg/ml. The pCR rate was 48.0% in patients with low SDF-1 expression and 24.4% in patients with high SDF-1 expression (P<0.001, **Figure 2B**). The correlation between the serum SDF-1 levels, TILs, and tumor regression (according to MP grades) is also shown in **Figures 2C, D**). Low levels of SDF-1 and high levels of TILs were significantly correlated with a relatively better response. For instance, the proportion of patients with poor response (MP 1, 2) was almost doubled in both high SDF-1 (22.2%, compared to 10.6% in low SDF-1) and low TILs (23.5%, compared to 9.8% in high TILs).

We also investigated the performance of SDF-1 at different TIL levels. **Supplementary Table 1** shows that the subgroup according to SDF-1 and TIL level (SDF-1 low/TILs-high, SDF-1 low/TILs-low, SDF-1 high/TILs-high and SDF-1 high/TILs-low) is independently correlated to pCR. Interestingly, the difference in the pCR rate between low and high SDF-1 levels was only significant in patients with high TILs (63.5% vs. 31.4%, P<0.001) but not in patients with low TILs (31.7% vs. 20.0%, P=0.089) (**Figure 2E**). The distribution of tumor regression also showed a similar phenomenon, indicating that patients with high TILs and low SDF-1 experienced a favorable response to NAC (**Figure 2F**).

We also analyzed the change in serum SDF-1 before and after NAC. The mean value of SDF-1 (pg/ml) in nonpCR patients was 380.9 (95% CI: 355.2-406.6) at baseline and 392.2 (95% CI: 361.6-422.7) at surgery, whereas the mean value of SDF-1 (pg/ml) in pCR patients was 288.6 (95% CI: 253.4-323.8) at baseline and 206.4 (95% CI: 184.1-228.7) at surgery. The reduction in SDF-1 before and after NAC was correlated with pathological response, as the mean reduction was -11.3 (95% CI: -47.4-24.5) (pg/ml) in nonpCR patients and 82.2 (95% CI: 49.7-114.8) (pg/ml) in pCR patients (P=0.004). In subgroup analyses, a significant difference in SDF-1 reduction between pCR and nonpCR responders was only observed in patients with high TILs (**Supplementary Figure 2**).

### SDF-1 and patient survival

For all patients in this study, the median follow-up time was 50 months. Among the 103 patients who achieved pCR, only 4 developed disease recurrence or metastasis. However, in the remaining 200 patients in the nonpCR group, 71 had cases of event or death (35.1%). Therefore, we developed survival analyses in only 200 nonpCR responders.

**Figure 3:**
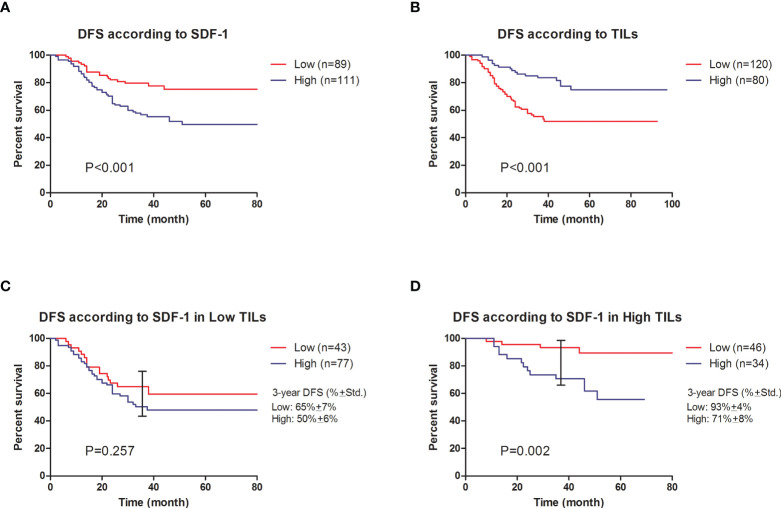
Cumulative disease-free survival of nonpCR patients after NAC. **(A)** DFS according to SDF-1 expression (log-rank test P<0.001). **(B)** DFS according to TIL expression (log-rank test P<0.001). **(C)** DFS according to SDF-1 expression in low TIL patients (log-rank test P=0.257. The observed 3-year DFS rates were 65% ± 7% and 50% ± 6% in the low SDF-1 and high SDF-1 groups, respectively. **(D)** DFS according to SDF-1 expression in high TIL patients (log-rank test P=0.002). The observed 3-year DFS rates were 93% ± 4% and 71% ± 8% in the low SDF-1 and high SDF-1 groups, respectively.

A Cox regression model was used to detect the prognostic biomarker in univariate and multivariate analyses (**Table 2**). Residual tumor size (P=0.018), residual involved nodes (P<0.001), tumor Ki-67 (P<0.001), serum SDF-1 at baseline (P=0.046), serum SDF-1 at surgery (P<0.001), and TILs at surgery (P<0.001) were significant predictors of DFS and entered the multivariate Cox regression model with forward selection. In the multivariate Cox regression model, both SDF-1 and TIL expression at surgery were independent predictors for DFS (SDF-1: P=0.011; HR=1.980, 95% CI: 1.170-3.350, low level as reference; TILs: P=0.012; HR=0.487, 95% CI: 0.278-0.855, low level as reference). However, serum SDF-1 at baseline failed to show independent prognostic value (P=0.559). The survival distribution by Kaplan–Meier survival curve is shown in **Figure 3**. Higher DFS was observed in nonpCR patients with low SDF-1 (**Figure 3A** Log-rank test P<0.001) and high TILs at surgery (**Figure 3B** Log-rank test P<0.001). We also demonstrated DFS according to SDF-1 expression in different subgroups of TIL levels (**Figures 3C, D**). Different levels of SDF-1 expression showed significant differences in the survival of patients with high TILs (log-rank test P=0.001), with 3-year DFS rates of 93% and 71% in patients with low SDF-1 and high SDF-1, respectively. However, SDF-1 expression failed to show prognostic value in patients with low TILs (log-rank test P=0.257), since patients in this subgroup had experienced similar unfavorable outcomes. The 3-year DFS was 65% and 50% in patients with low SDF-1 and high SDF-1, respectively.

**Table 2:** Univariate and multivariate survival analysis of non-pCR patients.

Factors	Disease-free survival		
	Univariate	Multivariate	
	P	P	HR (95% CI)
Age			
<40 vs. 40-60 vs.≥60	0.448	–	–
Menopausal status			
Pre vs. Post	0.350	–	–
Initial tumor status			
T2 vs. T3 vs. T4	0.163	–	–
Residual tumor size			
≤2cm vs. 2-5cm vs. >5cm	<0.001	0.018	Ref.
			0.802 (0.419-1.535)
			1.774 (0.993-3.168)
Residual involved nodes			
0 vs. 1-3 vs. ≥4	<0.001	<0.001	Ref. 0.862 (0.397-1.870) 2.685 (1.387-5.196)
Vascular invasion			
Negative vs. Positive	0.981	–	–
Grade			
I - II vs. III	0.051	–	–
Ki-67			
<20% vs.≥20%	<0.001	0.203	–
Serum SDF-1 at baseline			
Low vs. High	0.046	0.559	–
Serum SDF-1 at surgery			
Low vs. High	<0.001	0.011	Ref.
			1.980 (1.170-3.350)
CXCR4 at baseline			
- vs.+	0.111	–	–
CXCR4 at surgery			
- vs. +	0.188	–	–
TILs at baseline			
Low vs. High	0.218	–	–
TILs at surgery			
Low vs. High	<0.001	0.012	Ref 0.487 (0.278-0.855)

HR, hazard ratio; CI, confidence interval; SDF-1, stromal cell-derived factor-1; TILs, tumor-infiltrating lymphocytes; Ref., reference.

## Discussion

At present, the optimal chemotherapy regimen for TNBC remains controversial; therefore, it is managed with standard chemotherapy, including paclitaxel in combination with anthracycline or platinum drugs. Compared with other breast cancer subtypes, TNBC has a higher possibility of achieving pCR; however, this advantage is not clearly translated into improved overall survival due to the poor outcomes of nonpCR responders (21). Thus, it is important to identify sensitive responders. In recent studies, numerous biomarkers (e.g., tumor size, lymph node status, Ki-67, HER2, etc.) and imaging-based metrics (e.g., MRI and PET) have been studied for the prediction of pCR and survival (5, 6); however, reliable predictive and prognostic biological markers remain limited. In this study, we demonstrated that serum SDF-1 serves as a biomarker for predicting the treatment response and survival of TNBC patients who underwent NAC.

SDF-1 is a class of stromal cell-derived factors belonging to the chemokine CXC family systematically named CXCL12. It is a self-stable chemokine that marks CXCR4 and encodes a polypeptide of 89 amino acid residues (22). SDF-1 activates downstream signaling pathways, such as PAM and ERK1/2, and enhances cancer cell survival, proliferation, and chemotaxis by binding to its receptor CXCR4 (23). Previous studies have shown that high expression of SDF-1/CXCR4 is correlated with poor survival in various tumors, such as colorectal cancer (24), prostate cancer (25), pancreatic cancer (26), and breast cancer (27). However, limited data have reported the value of SDF-1/CXCR4 in predicting chemotherapy response. Karin Tamas et al. (28) reported that CXCR4 and SDF-1 are highly expressed in primary rectal tumors of patients presenting with metastatic disease, while radiochemotherapy and bevacizumab further upregulate CXCL12 expression; however, there were no differences in CXCR4 or CXCL12 expression at baseline between patients who had (n=9) vs did not have (n=30) a pCR. In contrast, Kim et al. (29) reported that unregulated expression of SDF-1α (P=0.016), after neoadjuvant chemoradiotherapy for rectal cancer, was significantly associated with treatment resistance. Our study presented new evidence that in TNBC, expression of SDF-1 in serum samples could identify chemosensitive tumors. To the best of our knowledge, no previous reports have identified the mechanism of acquiring chemotherapy resistance *via* upregulation of SDF-1 expression in breast cancer.

The expression of SDF-1 was significantly upregulated in myofibroblasts associated with invasive breast cancer compared with myofibroblasts obtained from normal breast tissue. Further evidence of SDF-1 production by stromal cells associated with breast cancer was provided by Orimo et al. (30). The mechanisms governing the stable regulation of SDF-1 in breast cancer-associated myofibroblasts have not been established; however, it is speculated that destruction of tumor cells by chemotherapeutic agents may release tumor-associated antigens, triggering an immune response that regulates SDF-1, which is particularly strong in patients whose immune systems are sensitive to certain tumor antigens before the onset of chemotherapy. Therefore, SDF-1 expression level might reflect the sensitivity of patients’ immune reaction to chemotherapy. It is also supported by our analysis that the reduction of SDF-1 was extremely high in pCR patients compared to nonpCR patients, indicating that regulation of SDF-1 was correlated with chemosensitivity.

Furthermore, we demonstrated that SDF-1 expression in patients with residual tumors was correlated with survival. It is suggested that residual chemotherapy-resistant disease after NAC is a substitute for chemotherapy-resistant micrometastases, which can eventually develop into clinically obvious metastatic breast cancer. Because TNBC is initially sensitive to NAC, residual tumors are generally more aggressive, which leads to poor prognosis and shorter RFS and OS (31, 32). In addition, some reports suggest that residual cancer cells in TNBC are a heterogeneous population, including subtypes with different outcomes (33). Therefore, the expression of SDF-1 in residual cancer cells may reflect a subtype of TNBC with stronger invasive behavior, leading to poor survival.

Interestingly, the predictive and prognostic value of SDF-1 was only significant in patients with high TILs but not in patients with low TILs, suggesting that TILs might play an important role in the interaction between SDF-1 and tumors. In recent years, many investigations have noted that TILs are important predictive and prognostic biomarkers in breast cancer patients. Carsten Denkert et al. (34) reported that the presence of tumor-associated lymphocytes in breast cancer is an independent predictor of response to anthracycline/taxane NAC in the cohort from the GeparDuo and GeparTrio study. Dieci et al. (17) also presented data that the presence of TILs in residual disease after NAC is associated with better prognosis in TNBC patients. These studies have led to the hypothesis that the pretreatment host response enhances the ability of chemotherapy to eliminate cancer cells, and the chemo-induced antitumor immune response might also influence patient survival (35). This hypothesis is strongly supported by our study, as TILs have also shown predictive and prognostic value in multivariate analyses. Since the SDF-1/CXCR4 axis plays a crucial role in recruiting immune cells such as MDSCs and Tregs to the tumor microenvironment (36, 37), we speculate that upregulation of SDF-1 expression may induce chemoresistance in TNBC *via* infiltration of immune cells. Further investigation of the relationship between SDF-1 and the precision subtyping of TILs is needed in our future study.

There are several limitations in this study. This was a retrospective study including 303 patients in single institution. Due to the relatively small sample size, we were not able to validate the cutoff values and establish a nomogram to predict pCR and survival. Additionally, the expression of SDF-1 was only detected at two time points, and it will be necessary to evaluate the change in SDF-1 at different periods of NAC, and the method used for choosing the cut-off point need to be further validated. It is also not clear at present whether our observation is restricted to NAC therapies in this study (paclitaxel and carboplatin) or may be a general feature of chemotherapy response.

In conclusion, the current study highlighted the utility of serum SDF-1 and established this as a potential predictive and prognostic marker in TNBC. We have presented that SDF-1, together with TILs, might help to identify patients who would benefit from chemotherapy and patients who need further intensified treatment strategies. Collectively, these biomarkers might help to shape preoperative and postoperative treatment strategies targeting SDF-1 and immune cells for the improvement of pCR rates and prevention of disease relapse in nonpCR patients.

## Data availability statement

The raw data supporting the conclusions of this article will be made available by the authors, without undue reservation.

## Ethics statement

The studies involving human participants were reviewed and approved by Shanghai Cancer Center Ethical Committee. The patients/participants provided their written informed consent to participate in this study.

## Author contributions

R-XW and SC contributed to the conception of the study, data analysis and interpretation, and writing the manuscript. PJ and YG made tissue sections and participated in ELISA and immunohistochemical analysis. SC and Z-MS contributed to the collection and assembly of data. All authors contributed to the article and approved the submitted version.

## Funding

This research was supported by the National Natural Science Foundation of China (81872134). The funders had no role in the study design, data collection and analysis, decision to publish, or preparation of the manuscript.

## Acknowledgments

The authors are grateful to Yin Zhou, Guang-Yu Liu, and Can-Ming Chen for their excellent data management.

## Conflict of interest

The authors declare that the research was conducted in the absence of any commercial or financial relationships that could be construed as a potential conflict of interest.

## Publisher’s note

All claims expressed in this article are solely those of the authors and do not necessarily represent those of their affiliated organizations, or those of the publisher, the editors and the reviewers. Any product that may be evaluated in this article, or claim that may be made by its manufacturer, is not guaranteed or endorsed by the publisher.

## Supplementary material

Supplementary Figure 1 Correlation between SDF-1 expression and other variables. SDF-1 expression was similar in patients with different ages, menopausal statuses, tumor stages, and Ki67 levels; however, high expression of SDF-1 was observed in patients with low levels of TILs (mean level 376.3 + 188.1 vs. 315.3 + 182.7, P=0.0048) and in patients with high expression of CXCR4 (mean level 295.4 + 161.3 vs. 383.6 + 195.7, P<0.001).

Supplementary Figure 2 Reduction in SDF-1 expression during NAC. The mean value of SDF-1 (pg/ml) in nonpCR patients was 380.9 (95% CI: 355.2-406.6) at baseline and 392.2 (95% CI: 361.6-422.7) at surgery, whereas the mean value of SDF-1 (pg/ml) in pCR patients was 288.6 (95% CI: 253.4-323.8) at baseline and 206.4 (95% CI: 184.1-228.7) at surgery. The reduction in SDF-1 before and after NAC was correlated with pathological response, as the mean reduction was -11.3 (95% CI: -47.4-24.5) (pg/ml) in nonpCR patients and 82.2 (95% CI: 49.7-114.8) (pg/ml) in pCR patients (P=0.004). In subgroup analyses, a significant difference in SDF-1 reduction between pCR and nonpCR responders was only observed in patients with high TILs.
