# Supplementary material for: SDF-1 expression and tumor-infiltrating lymphocytes identify clinical subtypes of triple-negative breast cancer with different responses to neoadjuvant chemotherapy and survival
Source: Front Immunol. 2022 Oct 20;13:940635. doi: 10.3389/fimmu.2022.940635 (PMC9630559; doi:10.3389/fimmu.2022.940635)
Supplement: Supplementary file 1 [file Table_1.docx]

**Supp. Table 1 pCR according to SDF-1/TILs subgroups**

| Subgroups | Number of patients (%) |  | Number of pCR (%) | Chi-Square P value | Multivariate* P value | Exp. OR  (95%CI) |
| --- | --- | --- | --- | --- | --- | --- |
|  |  |  |  | <0.001 | <0.001 |  |
| SDF-1 low/TILs-high | 63 (20.8) |  | 40 (63.5) |  |  | Ref. |
| SDF-1 low/TILs-low | 60 (19.8) |  | 19 (31.7) |  |  | 0.275 (0.128-0.571) |
| SDF-1 high/TILs-high | 70 (23.1) |  | 22 (31.4) |  |  | 0.277 (0.133-0.578) |
| SDF-1 high/TILs-low | 110 (36.3) |  | 22 (20.0) |  |  | 0.146 (0.072-0.296) |
|  |  |  |  |  |  |  |

*****

**Table 2 Univariate and multivariate survival analysis of non-pCR patients**

| **Factors** | **Disease-free survival** | | | |  |
| --- | --- | --- | --- | --- | --- |
|  | **Univariate** |  | **Multivariate** | |  |
|  | **P** |  | **P** | **HR (95% CI)** |  |
| Age |  |  |  |  |  |
| <40 vs. 40-60 vs.≥60 | 0.448 |  | - | - |  |
| Menopausal status |  |  |  |  |  |
| Pre vs. Post | 0.350 |  | - | - |  |
| Initial tumor status |  |  |  |  |  |
| T2 vs. T3 vs. T4 | 0.163 |  | - | - |  |
| Residual tumor size |  |  |  |  |  |
| ≤2cm vs. 2-5cm vs. >5cm | <0.001 |  | 0.018 | Ref. |  |
|  |  |  |  | 0.802 (0.419-1.535) |  |
|  |  |  |  | 1.774 (0.993-3.168) |  |
| Residual involved nodes |  |  |  |  |  |
| 0 vs. 1-3 vs. ≥4 | <0.001 |  | <0.001 | Ref.  0.862 (0.397-1.870)  2.685 (1.387-5.196) |  |
| Vascular invasion |  |  |  |  |  |
| Negative vs. Positive | 0.981 |  | - | - |  |
| Grade |  |  |  |  |  |
| I - II vs. III | 0.051 |  | - | - |  |
| Ki-67 |  |  |  |  |  |
| <20% vs.≥20% | <0.001 |  | 0.203 | - |  |
| Serum SDF-1 at baseline |  |  |  |  |  |
| Low vs. High | 0.046 |  | 0.559 | - |  |
| Serum SDF-1 at surgery |  |  |  |  |  |
| Low vs. High | <0.001 |  | 0.011 | Ref. |  |
|  |  |  |  | 1.980 (1.170-3.350) |  |
| CXCR4 at baseline |  |  |  |  |  |
| - vs.+ | 0.111 |  | - | - |  |
| CXCR4 at surgery |  |  |  |  |  |
| - vs. + | 0.188 |  | - | - |  |
| TILs at baseline |  |  |  |  |  |
| Low vs. High | 0.218 |  | - | - |  |
| TILs at surgery |  |  |  |  |  |
| Low vs. High | <0.001 |  | 0.012 | Ref  0.487 (0.278-0.855) |  |

Abbreviations: HR, hazard ratio; CI, [confidence](file:///D:\Program%20Files\Youdao\Dict\7.5.2.0\resultui\dict\?keyword=confidence)[interval](file:///D:\Program%20Files\Youdao\Dict\7.5.2.0\resultui\dict\?keyword=interval); SDF-1, stromal cell-derived factor-1; TILs, tumor-infiltrating lymphocytes; Ref., reference
