# Supplementary figures and images for: SDF-1 expression and tumor-infiltrating lymphocytes identify clinical subtypes of triple-negative breast cancer with different responses to neoadjuvant chemotherapy and survival
Source: Front Immunol. 2022 Oct 20;13:940635. doi: 10.3389/fimmu.2022.940635 (PMC9630559; doi:10.3389/fimmu.2022.940635)

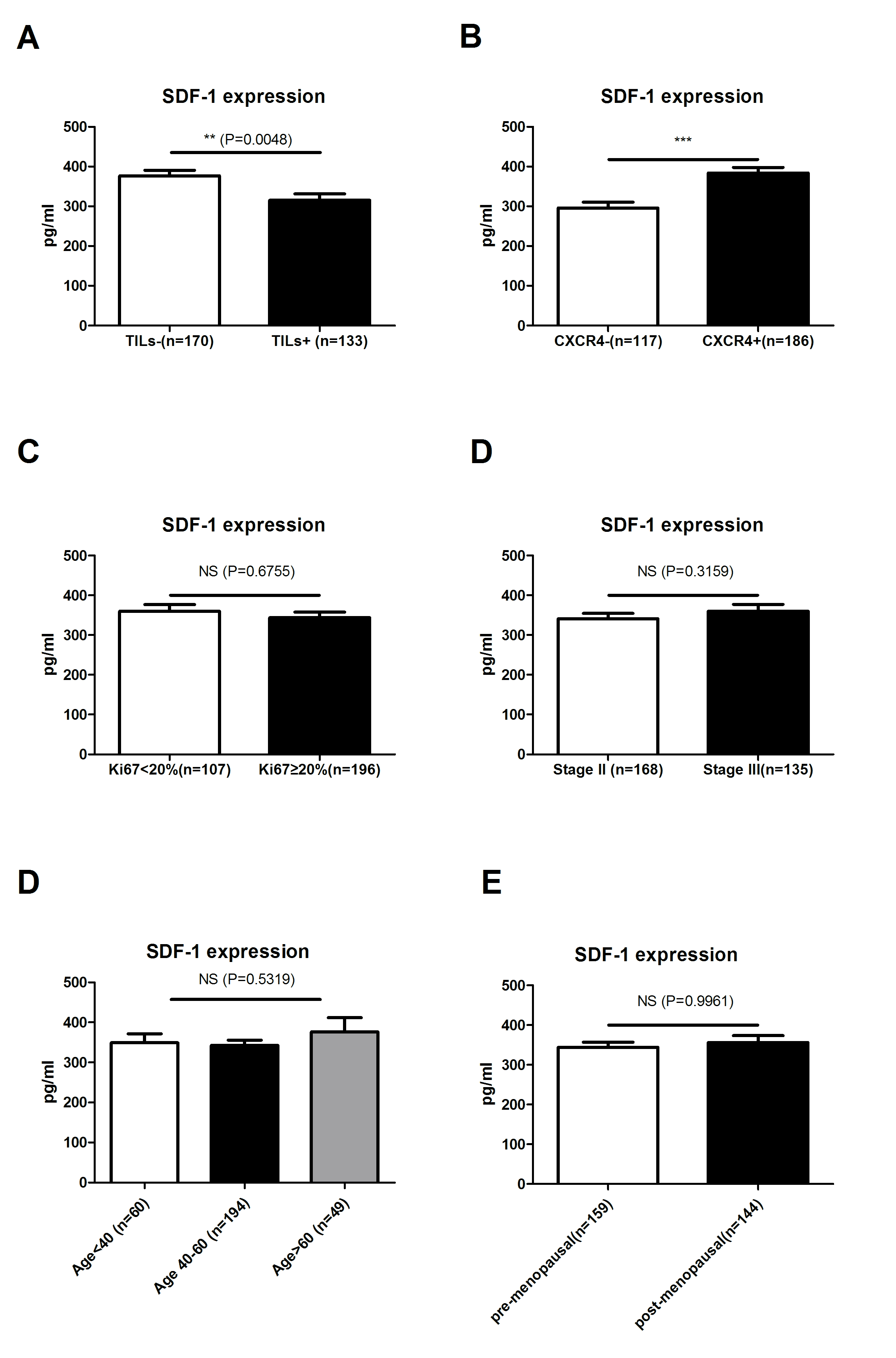

Supplement: Supplementary Figure 1 — Correlation between SDF-1 expression and other variables. SDF-1 expression was similar in patients with different ages, menopausal statuses, tumor stages, and Ki67 levels; however, high expression of SDF-1 was observed in patients with low levels of TILs (mean level 376.3 + 188.1 vs. 315.3 + 182.7, P=0.0048) and in patients with high expression of CXCR4 (mean level 295.4 + 161.3 vs. 383.6 + 195.7, P<0.001). [file Image_1.tif]

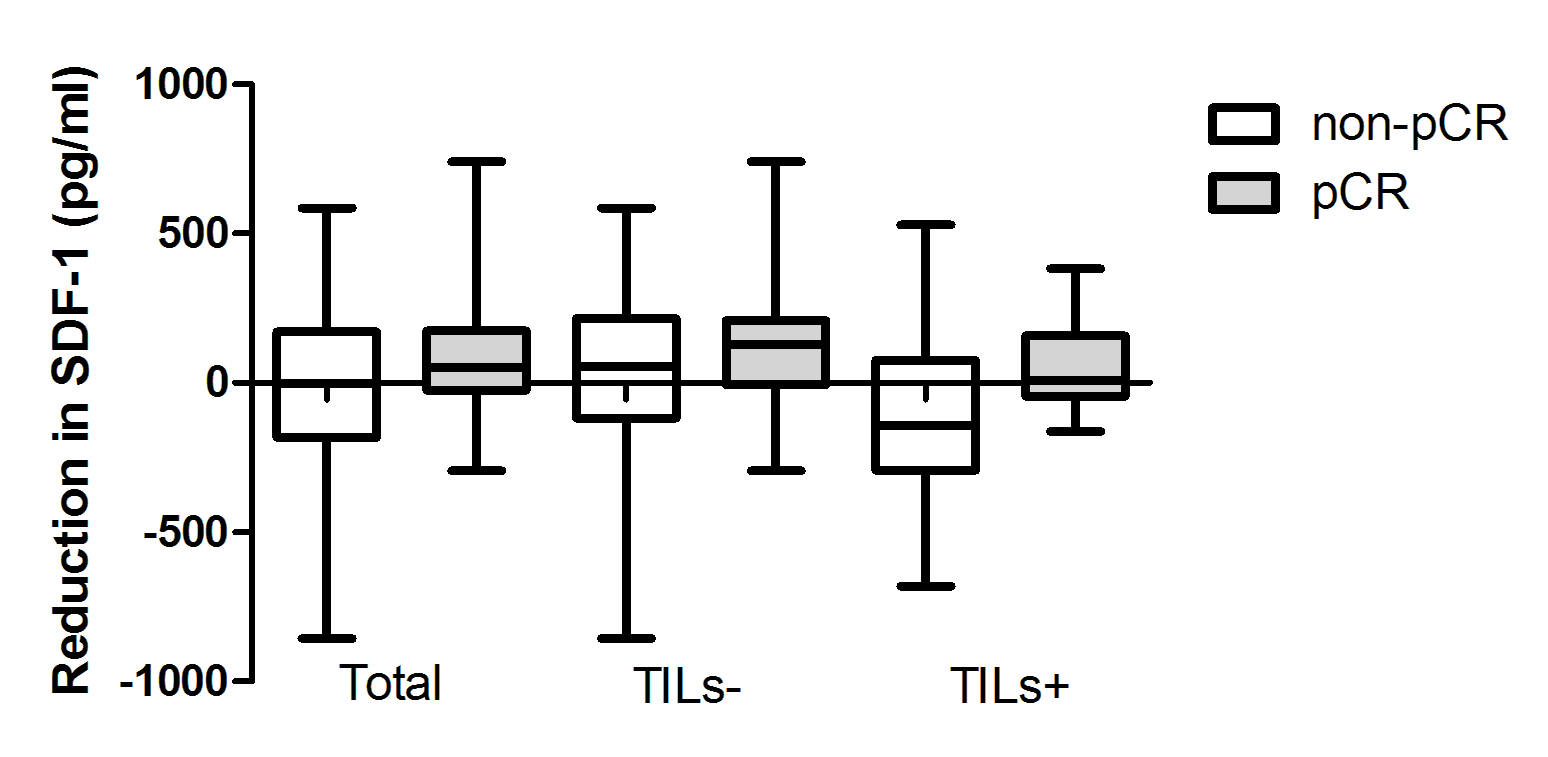

Supplement: Supplementary Figure 2 — Reduction in SDF-1 expression during NAC. The mean value of SDF-1 (pg/ml) in nonpCR patients was 380.9 (95% CI: 355.2-406.6) at baseline and 392.2 (95% CI: 361.6-422.7) at surgery, whereas the mean value of SDF-1 (pg/ml) in pCR patients was 288.6 (95% CI: 253.4-323.8) at baseline and 206.4 (95% CI: 184.1-228.7) at surgery. The reduction in SDF-1 before and after NAC was correlated with pathological response, as the mean reduction was -11.3 (95% CI: -47.4-24.5) (pg/ml) in nonpCR patients and 82.2 (95% CI: 49.7-114.8) (pg/ml) in pCR patients (P=0.004). In subgroup analyses, a significant difference in SDF-1 reduction between pCR and nonpCR responders was only observed in patients with high TILs. [file Image_2.tif]
